# A novel germline *BRCA2* mutation in a Chinese patient with prostate cancer sensitive to platinum chemotherapy: a case report

**DOI:** 10.1186/s12894-021-00879-4

**Published:** 2021-08-23

**Authors:** Lijuan Jiang, Zunguang Bai, Shoulun Zhu, Tingting Zhao, Yining Yang, Zhiyong Li, Dong Chen, Zhiming Wu, Yanjun Wang, Fangjian Zhou, Yonghong Li

**Affiliations:** 1 Department of Urology, Sun Yat-Sen University Cancer Center, Guangzhou, 510060 China; 2 Department of Urology, The Guangdong Provincial Hospital of Traditional Chinese Medicine, Guangzhou, 510000 China; 3 GloriousMed Clinical Laboratory (Shanghai) Co., Ltd., Shanghai, 200120 China

**Keywords:** Prostate cancer, BRCA2, Family history, Platinum chemotherapy

## Abstract

**Background:**

Germline *BRCA2* mutation is associated with an aggressive prostate cancer phenotype and indicates higher risk for hereditary cancer. Recently, numerous studies have attempted to identify the genomic landscape of prostate cancer to better understand the genomic drivers of this disease and look for the molecular targets to guide treatment selection.

**Case presentation:**

We report a 67-year-old patient diagnosed with prostate cancer who experienced rapid disease progression after androgen deprivation therapy and subsequent docetaxel treatment. The patient had a strong family history of malignancy as his mother was diagnosed with breast cancer and his father was died of lung cancer. Next generation sequencing demonstrated a novel pathogenic germline *BRCA2* mutation (p.Gly2181Glufs*10) in the patient. His mother with breast cancer and his son were found to have the same *BRCA2* mutation. The patient experienced impressive and durable responses to carboplatin treatment.

**Conclusions:**

This case demonstrated that the carboplatin could have a dramatic antitumor effect on patients with prostate cancer with germline *BRCA2* mutations and family history will help to ensure that patients and their families can be provided with proper genetic counseling.

## Background

Recently, numerous studies have attempted to identify the genomic landscape of prostate cancer (PCa) to better understand the genomic drivers of this disease and to identify molecular targets to guide treatment selection. About 8–12% of patients with metastatic PCa harbor germline aberrations in DNA damage repair (DDR) genes, including *BRCA1/2* and *ATM* [1, 2]. *BRCA2* mutations were detected in 3–5.3% of patients with PCa [1, 3, 4].

Deleterious germline mutations in *BRCA2* are known genetic events that confer increasing risk for patients with PCa [4, 5] and a germline *BRCA2* mutation is an independent prognostic factor for early-onset phenotype, aggressive disease, and poor clinical outcomes [4, 6, 7]. Furthermore, emerging evidence showed that DDR defects might predict sensitivity to poly-ADP ribose polymerase inhibitors and platinum agents in this molecular subtype of PCa [8–10].

Here, we report the case of a Chinese patient with metastatic castration-resistant prostate cancer (mCRPC) and a family history of cancers who experienced rapidly progressive disease. Importantly, this patient had a novel germline pathogenic mutation in *BRCA2* and subsequently showed a remarkable response to treatment with single platinum chemotherapy. Meanwhile, this mutation was confirmed as an inherited mutation from his mother, who had breast cancer and had been passed on to his son.

## Case presentation

**Fig. 1:**
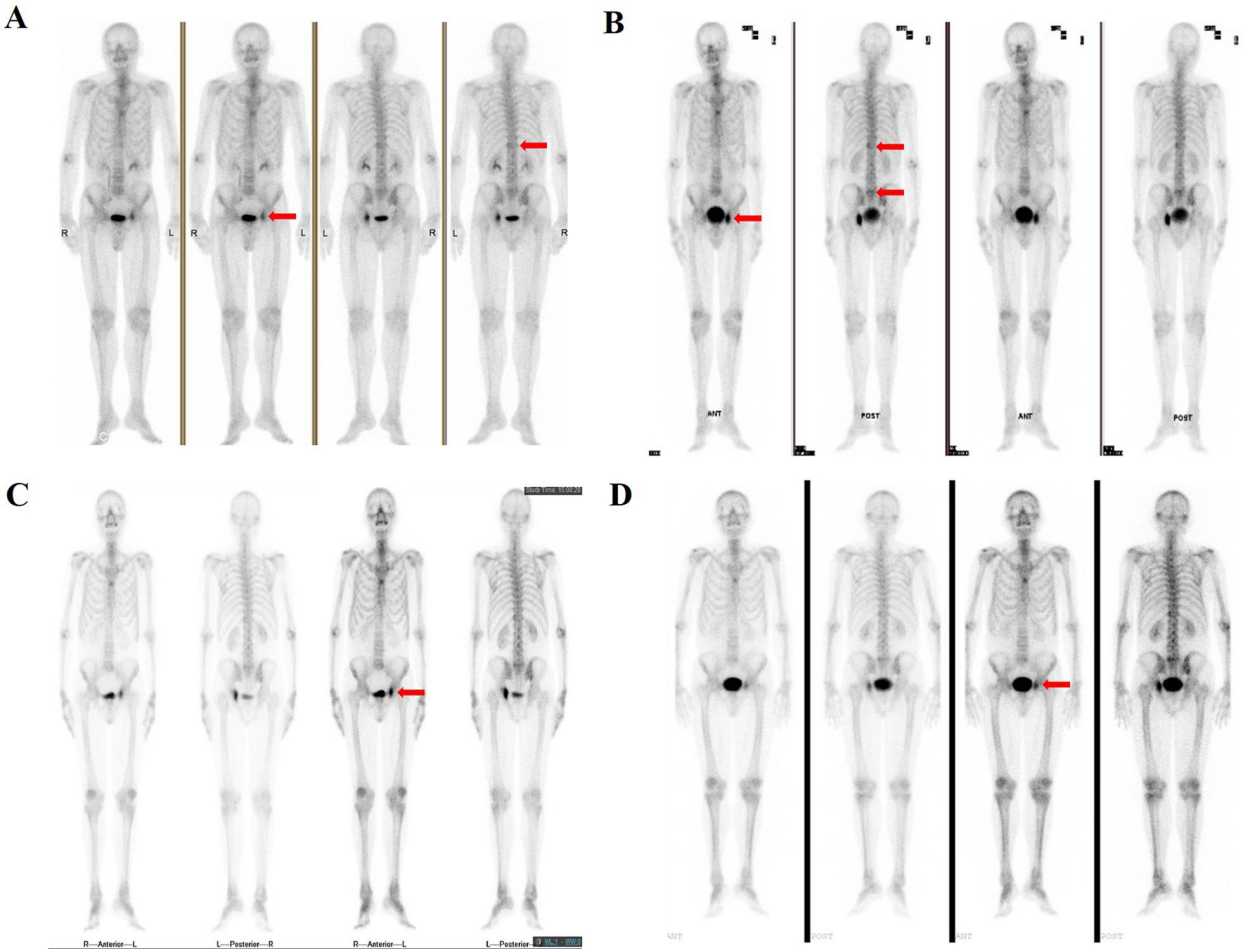
**A** Bone scan showing the bone metastatic lesions in left ilium and the 12th thoracic vertebra at diagnosis. **B** Ga-68 PSMA PET-CT showing multiple bone metastases lesions after 4 months of androgen deprivation therapy. **C** Bone scan showing one bone metastatic lesions in left ilium after six cycles of carboplatin chemotherapy. **D** Bone scan showing one bone metastatic lesions in left ilium on May 29th, 2020. The red arrow heads indicate the bone metastatic lesions of the patient. PET-CT: positron emission tomography-computed tomography

**Fig. 2:**
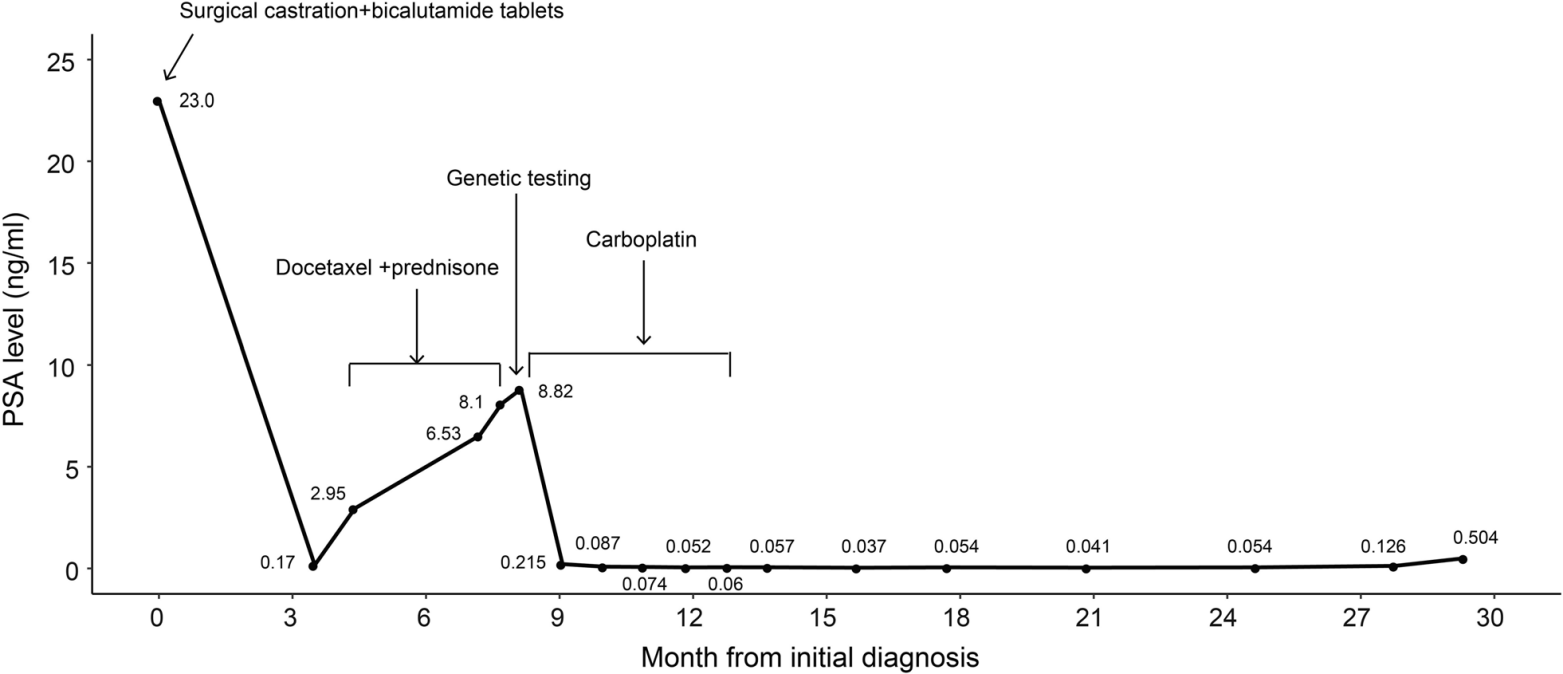
The clinical treatment course and prostate-specific antigen response

The patient was diagnosed at 67 years of age on May 18^th^, 2018, with an elevated prostate-specific antigen (PSA) level of 23 ng/ml. Prostate biopsy demonstrated adenocarcinoma with a Gleason score of 7 (4 + 3). Magnetic resonance imaging examination showed abnormal signals on both sides of the prostate. A bone scan showed two bone metastatic lesions (Fig. 1A). The clinical stage was evaluated as cT2N0M1b. The clinical treatment course of the patient is presented in Fig. 2.

The patient underwent bilateral orchidectomy on May 25th, 2018, and then achieved castrate levels of testosterone, followed by antiandrogen treatment with bicalutamide tablets 50 mg daily. His PSA level decreased to 0.17 ng/ml on August 29th, 2018. However, he developed castration-resistant disease after 4 months of androgen deprivation therapy with, during which his PSA level rose to 2.95 ng/ml and further examination showed multiple bone metastases (Fig. 1B, Fig. 2). The patient then changed to receive five cycles of docetaxel, while his PSA level continued to rise during this period. On January 15th, 2019, his PSA level increased to 8.82 ng/ml.

**Fig. 3:**
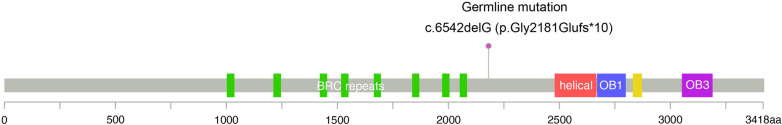
The germline mutation site in *BRCA2* of the patient. His mother and son shared the germline mutation

At this time, because he had a strong family history of malignancy (his mother was diagnosed with breast cancer and his father died of lung cancer), we performed next-generation sequencing (NGS) targeting 66 genes associated with PCa using the patient’s blood sample. The final report demonstrated that he had a germline *BRCA2* NM_000059.3: c.6542delG (p.Gly2181Glufs*10) mutation in exon 11 (Fig. 3), which had not been reported previously.

In light of the test result, the patient was subsequently turned into single agent carboplatin (AUC 5, 650 mg) treatment on January 15^th^, 2019, which led to a remarkable response (Fig. 2). After only one cycle of treatment, his PSA level was reduced from 8.82 to 0.215 ng/ml. At the third cycle, carboplatin was reduced to 550 mg because of grade 2 leukopenia. After sixth cycles of carboplatin chemotherapy, the PSA level declined to a nadir of 0.06 ng/ml and bone scan revealed one bone metastatic lesion in left ilium (Fig. 1C). Follow-up bone scan on May 29th, 2020 showed no imaging progression (Fig. 1D). On October 12th, 2020, 21 months after his first carboplatin chemotherapy, the patient remains well with low PSA levels (0.504 ng/ml) (Fig. 2).

Considering his family history (his mother was diagnosed with breast cancer at the age of 73 and his father died of lung cancer), his mother who was 90 years old and his 40-year-old son accepted genetic testing subsequently. As expected, the same *BRCA2* c.6542delG mutation was confirmed in both of them.

## Discussions and conclusions

As far as we know, this is the first report of a germline *BRCA2* c.6542delG mutation. It was located between the eighth BRC repeats and the DNA binding domain in BRCA2, which is predicted to result in premature termination of the BRCA2 protein (p.Gly2181Glufs*10). Besides, the genetic testing results of his mother and son revealed the same *BRCA2* mutation, suggesting that it could contribute to cancer development in this family. Taken together, this evidence strongly supports the view that this *BRCA2* mutation is pathogenic.

BRCA2 plays a critical role in homologous recombination DNA repair of double-strand breaks [11]. Deleterious *BRCA2* mutations are confirmed to increase the risk of developing lethal PCa and show a degree of familial aggregation. Patients with inherited *BRCA2* mutations present a more aggressive PCa phenotype, have a higher probability of nodal involvement and distant metastasis, and are more likely to die at an earlier age [6, 12]. For localized PCa, the presence of a *BRCA2* mutation confers a higher metastatic relapse rate and shorter cause-specific survival after radical treatment [7]. Germline *BRCA2* mutation carriers with mCRPC have 50% cause-specific survival compared with that of non-carriers [13].

For our patient, NGS testing revealed a germline pathogenic *BRCA2* mutation. He seemed to have a mild disease at initial diagnosis based on clinical characteristics, with a Gleason score of 7, PSA level of 23 ng/ml, and a state of oligometastasis. It had been indicated that patient with oligometastatic PCa may benefit from cytoreductive local treatment [14]. So we have planned to perform cytoreductive prostatectomy for him after 6 months of androgen deprivation therapy. However, he rapidly progressed to castration-resistance within four months. Then the cytoreductive prostatectomy was cancelled. Subsequently, he was treated with five cycles of docetaxel but the PSA level continued to rise. The rapid development of his disease confirmed *BRCA2* mutations are associated with poorer prognosis and ineffective routine treatment, as mentioned above.

Androgen deprivation therapy combined with bicalutamide was not routinely recommended for metastatic castration-sensitive PCa in international guidelines. However, this combined treatment was still used in some patients in China, since several retrospective studies and meta-analyses showed that this regimen may bring survival benefits to some patients with advanced PCa [15, 16].

Platinum, which is widely used in diverse cancers, induces crosslinking of DNA, thereby inhibiting DNA repair and DNA synthesis in cancer cells. Patients respond frequently to platinum. However, platinum agents are not recommended for routine use in the treatment of PCa, because a phase III trial of platinum compounds failed to demonstrate a significant overall survival benefit in unselected patients with mCRPC [17]. Nevertheless, increasing evidence suggests that a DNA repair defect has an impact on the sensitivity of platinum agents. Cheng et al*.* first reported that three patients with mCRPC with biallelic inactivation of *BRCA2* achieved an exceptional response to platinum chemotherapy [9]. Retrospective analysis of the relationship of pathogenic *BRCA2* germline variants and carboplatin-based chemotherapy in 141 men with mCRPC showed that *BRCA2* mutation carriers had a higher response rate to carboplatin-based chemotherapy than non-BRCA2 carriers [10]. More recently, another study presented three mCRPC cases with homologous recombination DNA repair defects, who experienced impressive and durable responses to carboplatin [18]. In our case, the patient started on single carboplatin treatment based on the NGS analysis of a pathogenic *BRCA2* germline mutation and long-term control of the disease was achieved. His response supported the view that BRCA2 could be a predictive biomarker for platinum response.

Family history is a strong risk factor for PCa. Hereditary mutations in *BRCA2* have been associated with increasing risk for PCa in various studies [4, 5, 19]. This patient was identified as having a germline pathogenic *BRCA2* mutation NM_000059.3: c.6542delG(p.Gly2181Glufs*10). This inherited mutation was later confirmed in his mother who had breast cancer and in his son, indicating high risk for PCa and breast cancer [20, 21]. Further monitoring via annual PSA screening was recommended for his son.

In conclusion, this case demonstrates that carboplatin could have a dramatic anti-tumor effect on patients with PCa with germline *BRCA2* mutations. The effect of synthetic lethality between DNA repair defects and platinum therapy has renewed interest in evaluating the role of platinum agents in the treatment of PCa. Our report supports the value of genetic testing for patients with PCa with mild clinical characteristics but with rapidly progressive disease, which could illuminate the genomic characteristics of the tumor, and provide implications for treatment strategy. In addition, incorporating the family history will help to ensure that patients and their families are provided with proper genetic counseling for effective cancer risk assessment and gain the most from the preventive benefits of genetic medicine.

## Data Availability

Not applicable.

## Abbreviations

PCa: Prostate cancer
DDR: DNA damage repair
mCRPC: Castration resistant prostate cancer
NGS: Next-generation sequencing
